# Silent bacteria: Intrinsic bioremediation potential in the sediments of Lake Faro (Messina, Italy)

**DOI:** 10.3934/microbiol.2026015

**Published:** 2026-06-22

**Authors:** Alessia Lunetta, Simone Cappello, Salvatore Giacobbe, Gabriella Caruso, Sabrina Patania, Antonietta Specchiulli, Tommaso Scirocco, Monia Renzi, Antonella D'Amore, Maria Genovese

**Affiliations:** 1 Department of Biological, Geological and Environmental Sciences (BIGEA), University of Bologna—Piazza di Porta S. Donato 1, 4126 Bologna (BO), Italy; 2 Institute for Biological Resources and Marine Biotechnologies (IRBIM) section of Messina, National Research Council (CNR-IRBIM), Spianata S. Raineri 86, 98122 Messina, Italy; 3 Institute of Polar Sciences, National Research Council (CNR-ISP), Spianata S. Raineri 86, 98122 Messina, Italy; 4 PhD School of “Applied Biology and Experimental Medicine”, Faculty of Sciences, University of Messina, Viale F. Stagno D'Alcontres 5, 98166 Messina, Italy; 5 Institute for Biological Resources and Marine Biotechnologies (IRBIM) section of Lesina, Na-tional Research Council (CNR-IRBIM), Via Pola 4, 71010 Lesina (Foggia), Italy; 6 University of Trieste, Via L. Giorgieri, 10, Italy

**Keywords:** bioremediation, hydrocarbon-degrading bacteria, lake sediments, microcosms, transitional ecosystem

## Abstract

Lake sediments can act as reservoirs for contaminants and host microbial communities with potential roles in natural ecosystem functioning. This study explored the occurrence of hydrocarbon-degrading bacteria, including “silent” hydrocarbon-responsive populations detectable after selective enrichment, in interfacial sediments of Lake Faro, a brackish meromictic basin in Messina, Italy. Sediment samples collected from five stations were chemically characterized and incubated in microcosms enriched with tetradecane, phenanthrene, or crude oil as the sole carbon and energy source. Chemical analyses revealed that hydrocarbons HCs > 12 were below the quantification limit at all stations, while PAHs were detectable at low concentrations. After 80 days of enrichment, microbial abundance increased, especially in tetradecane and crude oil–amended microcosms, and hydrocarbon-degrading bacteria were selectively enriched. Overall, 25 bacterial isolates were obtained, of which 16 were identified by 16S rRNA gene sequencing and assigned to taxa with reported hydrocarbon-degrading potential, including *Isoalcanivorax pacificus*, *Marinobacter hydrocarbonoclasticus*, *Pseudoalteromonas* sp., *Vibirio alginolyticus*, and *Stappia indica*. Several strains showed emulsifying activity, with E_24_ values up to 60%. These findings suggest that Lake Faro sediments host a latent hydrocarbon-responsive bacterial fraction, with intrinsic bioremediation potential and ecological relevance for natural attenuation processes in transitional aquatic ecosystems.

## 1. Introduction

Transitional environments represent essential components of the global aquatic ecosystem, acting as interfaces between terrestrial and marine systems. They are characterized by unique physicochemical properties that host multi-kingdom microbial communities (bacteria, archaea, fungi, viruses, etc.), which in turn form complex ecological networks through competition, symbiosis, and cooperation [1],[2]. These microbial assemblages represent the core of biogeochemical processes. Bacteria and archaea play a key role in the mineralization of organic matter, nutrient transformation, and the regulation of metabolic fluxes of greenhouse gases, such as methane (CH_4_) [3]; fungi, in contrast, are specialized in the degradation of highly recalcitrant compounds, such as lignin, thereby promoting more efficient recycling of organic matter [4]. Within these transitional systems, sediments emerge as a pivotal compartment where microbial diversity, organic matter turnover, and biogeochemical transformations converge, concentrating both nutrients and contaminants and thereby amplifying their ecological relevance [5]–[7]. Acting as dynamic reservoirs of nutrients and organic carbon, sediments facilitate continuous exchanges with the overlying water column, thereby tightly coupling benthic and pelagic processes [8]. The rapid growth of the human population and intense anthropogenic activities contribute to increasing human pressure on these systems [9]–[11]. Pollutants such as metals [12], pesticides [13], polychlorinated biphenyls (PCBs) [14], and polycyclic aromatic hydrocarbons (PAHs) [15], owing to their hydrophobic nature and environmental persistence [16], tend to bind to suspended particles and accumulate in sediments [17]. Consequently, sediments act as sinks for chemical pollutants and can be regarded as environmental archives that record the temporal history of contamination [17]. Contamination levels in the uppermost sediment layers (0–5 cm) are directly influenced by the occurrence and spatial distribution (point or diffuse) of pollution sources whose wastes or effluents are released into the environment, thereby impacting bacterial communities [17]. Numerous studies have investigated the occurrence, fate, and dynamics of pollutants in these environments. Seminal work by Mitsch et al. [18] comprehensively reviewed wetland ecological functions, including pollutant filtration and retention processes, while Zedler and Kercher [19] highlighted the capacity of wetlands to buffer contaminants and restore water quality. In this context, microorganisms play a key role in the biodegradation of contaminants that accumulate in sediments through particle association or sedimentation processes, thereby influencing pollutant fate and overall ecosystem health [20],[21]. Characterizing the sediment microbiome and its intrinsic biodegradation potential, therefore, provides valuable insights into pollutant impacts on benthic functioning and ecosystem-level processes [22]. In particular, hydrocarbon-degrading bacteria have been widely documented in aquatic and sedimentary environments, where they contribute to the natural attenuation of petroleum-derived compounds by degrading aliphatic and aromatic hydrocarbons [23],[24]. These microorganisms often occur as complex consortia, including genera such as *Pseudomonas*, *Alcanivorax*, *Bacillus*, and *Marinobacter*, capable of degrading a broad spectrum of aliphatic and aromatic hydrocarbons through specialized enzymatic pathways [25],[26]. While extensive research has focused on marine sediments and heavily contaminated systems, demonstrating high taxonomic and functional diversity of hydrocarbonoclastic communities [27],[28], comparatively fewer studies have addressed freshwater and lacustrine sediments, especially under low-level or chronic contamination conditions [29],[30]. Moreover, the ecological regulation of these bacterial assemblages, their coupling with sediment biogeochemical cycles (e.g., nitrogen and sulfur transformations), and the activation mechanisms of latent or “silent” hydrocarbon-degrading populations remain poorly understood [24],[31]. These knowledge gaps limit our ability to accurately evaluate the intrinsic bioremediation potential of lake sediments and to predict microbial responses to hydrocarbon inputs under changing environmental conditions. Within this framework, “silent bacteria” refers here to latent hydrocarbon-responsive bacteria that may remain functionally undetected under natural, non-enriched conditions but become detectable after selective enrichment with hydrocarbons. This interpretation is consistent with the broader ecological concept of microbial seed banks, in which rare or dormant microbial populations may persist in the environment and respond when suitable substrates or environmental conditions become available [32]. Based on these considerations, the present study focused on Lake Faro, a brackish meromictic lake, whose physicochemical characteristics and hydrological connection make it a suitable site for exploring and evaluating the intrinsic hydrocarbon-responsive potential of sediment-associated bacteria. Exploring for the first time the presence of hydrocarbon-degrading bacteria within the microbial benthic community of Lake Faro, this study aimed to (i) analyze whether hydrocarbons pollute the sediments of Lake Faro, (ii) assess phylogenetically the native microbial community hosted in this environmental matrix, (iii) monitor changes in bacterial community structure and composition in experimental microcosms amended with various hydrocarbons, and (iv) identify bacterial strains, including those potentially silent, playing a functional role in environmental restoration.

## 2. Materials and methods

### 2.1. Study area

**Figure 1.**
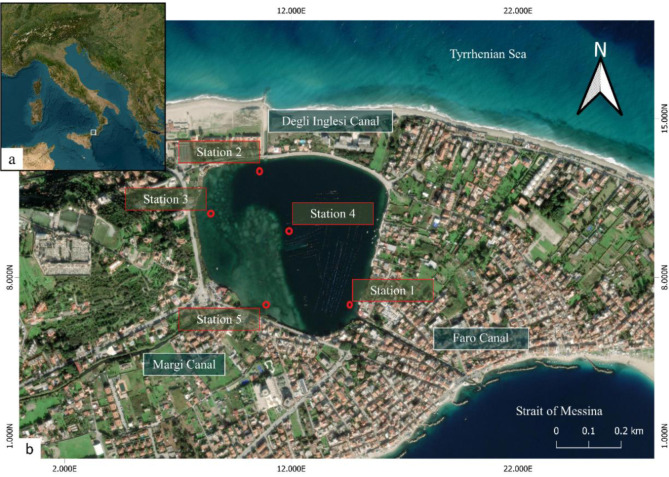
Study area. (a) Geographical location of the study site in Italy. (b) Satellite image of Lake Faro (Messina, Sicily) showing the sampling stations.

Lake Faro (38°16′07″ N, 15°38′13″ E), covering a surface of only 263,600 m^2^, is the deepest coastal basin in Italy, reaching 29 m depth in its eastern funnel-shaped area and only 3.5 m in its western flat portion (Figure 1). The lake is connected to the Strait of Messina by the “Faro” Canal, while it receives water from the Tyrrhenian Sea through the “Inglesi” Canal only in a short summer period. A connection with the nearby brackish Lake Ganzirri is provided by the “Margi” Canal [33]. Lake Faro is a brackish meromictic basin with an oxygenated mixolimnion that rarely exceeds 15 m in depth and a deeper anoxic sulfide-rich monimolimnion [34]. A peculiar “red water” chemocline generated by the action of sulfur photo-trophic bacteria [35] is present in Lake Faro. Here, a vertical zonation, with the mixolimnion reaching up to a 10-m depth and a persistent bottom stagnant layer, has been previously highlighted [36].

### 2.2. Sampling

Sediment samples were collected in November 2024 from five stations, almost homogeneously distributed in Lake Faro, excluding the depth anoxic zone (Figure 1 and Table 1). Sampling was standardized at approximately 2 m depth at all stations, corresponding to shallow oxygenated sediments, in order to reduce variability associated with the meromitic stratification of the lake and with local site-specific conditions, such as proximity to canals or human activities. At each station, sediments were collected using a small Van Veen crab (15 × 25 cm surface, 2 L volume). All samples, collected in duplicate, were quickly transported in a refrigerated box (4 ± 1 °C) for laboratory processing.

**Table 1.** Location of Lake Faro sampling stations (geographic coordinates) and sediment typology.

Station	Coordinates	Type of sediment	Reference
AL-01	38°16′00.4″N; 15°38′20.2″E	Muddy biodetritic	[37]
AL-02	38°16′15.1″N; 15°38′08.6″E	Slightly silty sand	[37]
AL-03	38°16′15.1″N; 15°38′08.6″E	Slightly muddy, gravelly sand	[37]
AL-04	38°16′06.4″N; 15°38′11.9″E	Sandy	[37]
AL-05	38°27′09.4″N; 15°63′58.4″E	Slightly silty, bioclastic sand	[37]

### 2.3. Environmental physical-chemical characterization

Concurrently with sediment sampling, the main chemical-physical variables [Temperature (T, °C), pH, salinity (PSU), atmospheric pressure (atm), and dissolved oxygen (D.O.)] were measured using a portable multiparameter probe (YSI multiparameter portable, Professional Plus).

### 2.4. Analysis of hydrocarbon concentrations and characterization

For chemical determinations, sediments were homogenized, freeze-dried, and successively sieved to remove particles >2 mm. Their chemical characterization (qualitative and quantitative analysis of hydrocarbons) was conducted by extraction, and polycyclic aromatic hydrocarbons (PAHs) and total hydrocarbons were determined by high-pressure liquid chromatography with photo diode array (HPLC-PDA) and infra-red spectroscopy (micro-FTIR). Ultrasonication was performed using acetone/hexane, and purification was performed by silica gel column chromatography according to Kumar et al. [38]. Regarding PAHs, 16 compounds defined as high-priority pollutants by US EPA were considered, namely naphthalene (Naph), acenaphthene (Ace), acenaphthylene (Acy), phenanthrene (Phe), anthracene (Anthr), fluoranthene (Fluo), fluorene (Fl), benz[a] anthracene (BaA), chrysene (Chry), benz[b] fluoranthene (BbF), benz[k]fluoranthene (BkF), benzo[a]pyrene (BaP), benzo(g,h,i)perylene (B.g.h.i.P), dibenz[a,h]anthracene (D.B.a.h.A), fluorene (fl), indeno[1,2,3-cd] (IND), and pyrene (Pyr).

### 2.5. Microbial abundance in natural samples

To monitor the abundance of microbial communities in the sediment, total prokaryotic counts (DAPI), cultivable bacteria (CFU), and most probable number (MPN) analysis were carried out.

### 2.6. Bacterial dislodging

From each sample, 1 g of sediment was placed in Bushnell Haas medium (9 mL of BH; Difco™ Bushnell-Haas Broth, BD Becton, Dickinson and Company) supplemented with 2% NaCl [39]. The samples were mixed by vortexing (MIX ARGOlab Vortex Mixer, Giorgio Bormac) at maximum speed for 30 s, placed in the ultrasonic cleaning bath (Branson 1200 Ultrasonic Cleaner, Branson) for bacterial dispersion from the sediment (3 min), and finally placed on ice for 3 min. This procedure was repeated three times. The samples were left to incubate at 25 ± 1 °C, and after precipitation processes, the supernatant was removed and used for direct counting.

### 2.7. Total bacterial counts (DAPI)

After dislodging treatment, microbial abundance was measured by the direct counting method (4′,6-diamidino-2-phenylindole, DAPI) [40] on samples obtained from sediment after the previously indicated dislodging. Cell counts were performed with DAPI (Sigma-Aldrich SrL, Milan, Italy) on formaldehyde-fixed samples (final concentration 2%). The sample was thus filtered on Nuclepore black polycarbonate filters (pore size 0.2 mm) according to standard analytical protocols [41]. The specimen slides were examined with a Zeiss Axioplan 2 Imaging Epifluorescence Microscope (Zeiss; Carl Zeiss Inc., Thornwood, N.Y.), and the labeled cells present in at least 30 microscopic fields were counted. All results were expressed as the number of cells per gram of wet sediment.

### 2.8. Cultivable bacteria (CFU)

The total active heterotrophic fraction was estimated by spreading 100 µL of 10-fold dilutions on Marine Agar 2216 medium (Difco S.p.a., Milan, Italy). All plates were incubated at 25 ± 1 °C for 20 days. The results were expressed as colony forming units (CFU) per gram of wet sediment [42].

### 2.9. Hydrocarbon-degrading bacterial count (MPN)

Most probable number (MPN) was determined to quantify heterotrophic and hydrocarbon-degrading bacteria. Sterile 96-well microplates (300 µL each), containing 270 µL of sterile Bushnell Haas medium (BH; Difco™ Bushnell-Haas Broth, BD Becton, Dickinson and Company) supplemented with 2% NaCl, were used [43]. A serial dilution was made in BH medium. The wells were inoculated by pipetting 30 µL of the sample. After sample inoculation, 1 µL of oil (Arabian Light Crude Oil), sterilized by filtration through a 0.2 µm syringe filter (Sartorius), was added to each well. The plates were incubated at 25 ± 1 °C for 20 days. After incubation, visual assessment of microbial load was performed according to the number of positive tubes as indicated by the American Public Health Association [44].

### 2.10. Microcosm setup

Microcosms were set up in sterile flasks (50 mL volume) filled with 25 mL of ONR7a mineral medium [45] and 1 g of sediment sample. ONR7a contained (per liter of distilled water) 40 g of NaCl, 11.18 g of MgCl_2_·6H_2_O, 3.98 g of Na_2_SO_4_, 1.46 g of CaCl_2_·2H_2_O, 1.3 g of TAPS0 {3-[N tris (hydroxymethyl) methylamino]-2 hydroxypropane sulfonic acid}, 0.72 g of KCl, 0.27 g of NH_4_Cl, 89 mg of Na_2_HPO_4_·7H_2_O, 83 mg of NaBr, 31 mg of NaHCO_3_, 27 mg of H_3_BO_3_, 24 mg of SrCl_2_·6H_2_O, 2.6 mg of NaF, and 2 mg of FeCl_2_·4H_2_O. To convert the liquid medium into a solid phase, bacterial agar (15 g L^−1^) was added to the solution [46]. Tetradecane [0.4% (v/v)], phenanthrene [0.4% (w/vol)], and crude oil [(0.4% (v/v); Arabian Light Crude Oil] were used as the sole source of carbon and energy because they represent, respectively, an aliphatic hydrocarbon, an aromatic polycyclic hydrocarbon, and a complex hydrocarbon mixture commonly associated with petroleum contamination [47]. This choice allowed the evaluation of the metabolic versatility and hydrocarbon-degrading potential of the bacterial isolates. All contaminants were sterilized before use. Microcosms enriched with tetradecane, phenanthrene, and oil were identified, respectively, as “TET”, “PHEN”, and “OIL”. One microcosm, indicated as “CONTROL”, without the presence of a carbon source, was also set up. All systems were incubated at 25 ± 1 °C for 80 days under shaking (Certomat IS B. Braun Biotech International).

### 2.11. Microbial abundance in experimental microcosms

After the incubation period (80 days), microbial abundance was estimated in the three microcosms by DAPI, CFU, and MPN methods, as previously reported.

### 2.12. Isolation of bacterial strains

From each enriched microcosm (TET, PHEN, and OIL), 100 µL of sample was spread on ONR7a agar plates (added with different hydrocarbon sources) and incubated at 25 ± 1 °C for 10 days. After growth, morphologically distinct bacterial colonies were isolated by repeated streaking on fresh ONR7a agar plates incubated at 25 ± 1 °C for an additional 10 days. Bacterial strains were stored at −20 °C in stock medium added with glycerol for further characterization [48].

### 2.13. Taxonomical characterization (16S rDNA sequencing)

Genomic DNA was extracted from each isolate using the DNeasy Blood & Tissue kit (QIAGEN, Germany) according to the manufacturer's instructions. Using a NanoDrop ND-1000 spectrophotometer (NanoDrop Technologies, Thermo Fisher Scientific, USA), the quality and concentration of the extracted DNA were quantified. Microbial DNA was amplified through polymerase chain reaction (PCR) using the Taq PCR Master Mix Kit (QIAGEN, Germany) and universal primers (27F 5′-AGAGTTTGATCCTGGCTCAG-3′ and 1492R 5′-TACGGYTACCTTGTTACGACT-3′) [39]. The amplification reaction was carried out in a total volume of 30 µL consisting of Taq PCR Master Mix 2× (15 µL); 10× primer mix (2 µM of each primer) (3 µL), and 11 µL of RNAse-free water. The PCR program consisted of 35 cycles with an initial 3 min hot start at 95 °C, followed by 30 cycles of 1 min at 94 °C, 1 min at 50 °C, and 2 min at 72 °C, and a final extension of 10 min at 72 °C. Purification and sequencing (Sanger method) were performed by Macrogen Inc. (Amsterdam, The Netherlands) using only the reverse primer (1492R). The 16S rRNA gene sequences of the closest relatives were identified with the Basic Local Alignment Search Tool (BLAST), provided by the National Center for Biotechnology Information (NCBI, Bethesda, MA, USA) [49]. Taxonomic assignments were based on BLAST similarity and phylogenetic placement. Species-level identification was retained only when supported by high sequence similarity and consistent phylogenetic affiliation. Phylogenetic trees with bootstrap values (1000 resampled datasets) were reconstructed using the MEGA 5 software [50] based on neighbor-joining (NJ), maximum parsimony (MP), and maximum likelihood (ML) algorithms.

### 2.14. Emulsifying activity (%, E_24_)

The emulsifying activity (%, E_24_) of the bacterial isolates derived from the different microcosms was evaluated by adding n-hexadecane as a model hydrophobic substrate. This substrate was selected because it is widely used in emulsification index assays as a standardized hydrocarbon phase to assess the ability of microbial metabolites to stabilize oil–water emulsions [51]. Therefore, the E_24_ assay was used as an indirect indicator of emulsifying/biosurfactant-like activity rather than as a direct measurement of hydrocarbon degradation. Cell-free culture broth and n-hexadecane were mixed at equal volumes by vortexing for 2 min, and the mixture was kept undisturbed for 24 h [51]. The emulsifying activity (%, E_24_) was calculated by measuring the height of the emulsified layer (hE) and the total height of the mixture (hT) present inside the test tube and applying the formula (Eq 1):

$${\text{E}}_{\text{24}}\%=\text{hE}/\text{hT}\times \text{1}00$$ (1)

### 2.15. Statistical analysis

Quantitative microbial abundance data, including total prokaryotic counts (DAPI), cultivable bacteria (CFU), and hydrocarbon-degrading bacteria estimated by the most probable number method (MPN), were expressed as mean ± standard deviation of duplicate measurements. For inferential analyses, station-level mean values were log10-transformed and used to compare microbial abundance among natural sediments and hydrocarbon-enriched microcosms. One-way analysis of variance (ANOVA), followed by Tukey's HSD post hoc test [52], was performed separately for DAPI, CFU, and MPN data. Differences were considered statistically significant at p < 0.05.

## 3. Results

### 3.1. Physical-chemical variables

The environmental variables measured at each station (Table 2) showed a homogenous temperature (26.1–26.2 °C) except for St. AL-02, where the temperature value was appreciably lower (25.7 °C). Slight differences were observed in pH values, which ranged from 6.59 (AL-01) to 7.09 (AL-05). Just like temperature, salinity also showed only one value significantly lower (27.34 in AL-03) compared to the narrow range of values (36.21–36.46) recorded at all other stations. Quite similar values of atmospheric pressure were measured. DO values were comprised between 5.5 and 6.6 mg/L.

**Table 2.** Mean values of the main chemical-physical parameters characterizing the study stations. D, depth; T, temperature; pH; S, salinity; atm, atmospheric pressure; DO, dissolved oxygen.

Station	D (m ± 0.1 cm)	T (±0.5 °C)	pH (±0.2)	S (PSU)	atm (mb ± 0.001)	DO (mg/L ± 0.2)
AL-01	2.4	26.1	6.59	36.21	1003.9	6.2
AL-02	2.5	25.7	6.75	36.38	1003.8	5.5
AL-03	2.3	26.0	6.93	27.34	1002.9	5.6
AL-04	2.5	26.2	6.73	36.46	1003.9	6.6
AL-05	2.4	26.2	7.09	36.43	1003.8	6.5

### 3.2. Concentrations of hydrocarbons

**Figure 2.**
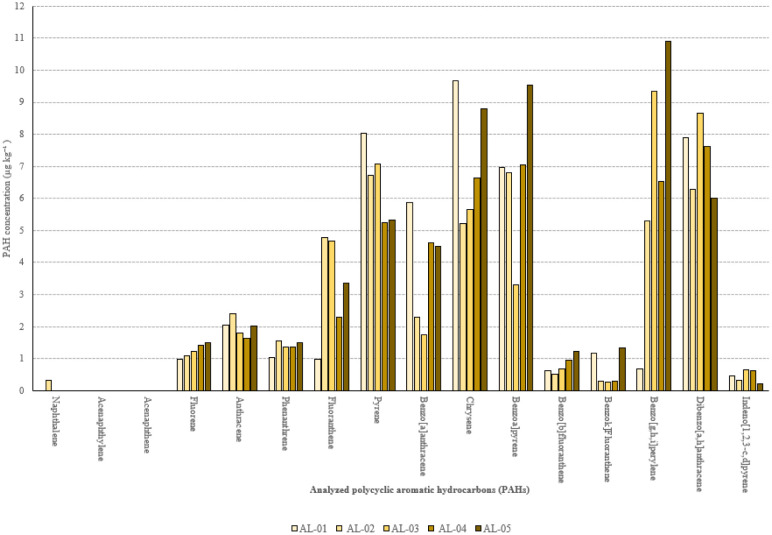
Presence (µg/Kg) and distribution of the main PAHs detected at the different examined stations.

The concentrations of n-alkanes (C > 12) and total PAHs measured in the sediment samples are reported in Table 3 and Figure 2.

**Table 3.** Hydrocarbon concentrations (HCs > 12 and PAHs) evaluated at each sampling station.

Station	HCs > 12 (µg/kg)	PAHs (µg/kg)
AL-01	<0.1	46.4
AL-02	<0.1	43.9
AL-03	<0.1	46.5
AL-04	<0.1	46.3
AL-05	<0.1	56.2

HCs > 12 were below the quantification limit (<0.1 µg kg^−1^) at all sampling stations, whereas total PAHs were detectable at low concentrations, ranging from 43.9 µg kg^−1^ at AL-02 to 56.2 µg kg^−1^ at AL-05. The highest total PAH value was observed at AL-05, located close to the mouth of the Margi Canal and near a shellfish farming area. AL-01, located near another farming area but also close to the Faro Canal, showed PAH concentrations comparable to those measured at AL-02, AL-03, and AL-04. Overall, these results indicate the absence of detectable HCs > 12 in the analyzed sediments, together with the occurrence of low but measurable PAH concentrations.

### 3.3. Microbial abundance in natural samples

The abundance of total prokaryotic cells, cultivable heterotrophic bacteria, and hydrocarbon-degrading bacteria in the natural sediment samples collected from Lake Faro was estimated by DAPI, CFU, and MPN analyses, respectively. These values, corresponding to the initial conditions used for the subsequent microcosm experiments, are reported in Table 4 as mean ± standard deviation of duplicate measurements.

**Table 4.** Microbial abundance measured at T_0_ in natural sediment samples and at T_80_ in sediment microcosms amended with tetradecane (TET), phenanthrene (PHEN), or crude oil (OIL). DAPI values are expressed as cells per gram of wet sediment, CFU values as CFU per gram of wet sediment, and MPN values as MPN per gram of wet sediment. Values are reported as mean ± standard deviation of duplicate measurements. Different superscript letters within each microbial parameter indicate significant differences among treatments based on one-way ANOVA followed by Tukey's HSD test performed on log10-transformed station-level mean values (p < 0.05).

			Isolate				
Parameter	Time	Treatment	AL-01	AL-02	AL-03	AL-04	AL-05
DAPI	T_0_	Natural sediment^a^	(2.75 ± 0.35) × 10^3^	(2.60 ± 0.14) × 10^3^	(2.45 ± 0.07) × 10^3^	(2.30 ± 0.14) × 10^3^	(2.95 ± 0.07) × 10^3^
	T_80_	TET^c^	(4.10 ± 0.14) × 10^3^	(4.30 ± 0.14) × 10^3^	(4.50 ± 0.14) × 10^3^	(4.90 ± 0.14) × 10^3^	(4.60 ± 0.14) × 10^3^
		PHEN^b^	(2.95 ± 0.07) × 10^3^	(3.25 ± 0.07) × 10^3^	(3.20 ± 0.14) × 10^3^	(3.10 ± 0.14) × 10^3^	(3.15 ± 0.07) × 10^3^
		OIL^b^	(3.15 ± 0.07) × 10^3^	(3.45 ± 0.07) × 10^3^	(3.55 ± 0.21) × 10^3^	(3.65 ± 0.07) × 10^3^	(3.65 ± 0.21) × 10^3^
CFU	T_0_	Natural sediment^a^	(52.0 ± 1.4) × 10^3^	(43.5 ± 3.5) × 10^3^	(43.0 ± 1.4) × 10^3^	(57.0 ± 1.4) × 10^3^	(38.5 ± 0.7) × 10^3^
	T_80_	TET^c^	(33.5 ± 0.7) × 10^3^	(34.5 ± 0.7) × 10^3^	(32.0 ± 1.4) × 10^3^	(43.0 ± 2.8) × 10^3^	(66.5 ± 2.1) × 10^3^
		PHEN^b^	(50.0 ± 1.4) × 10^3^	(45.0 ± 1.4) × 10^3^	(72.0 ± 1.4) × 10^3^	(65.5 ± 0.7) × 10^3^	(52.0 ± 1.4) × 10^3^
		OIL^b^	(73.0 ± 1.4) × 10^3^	(54.5 ± 0.7) × 10^3^	(65.5 ± 2.1) × 10^3^	(50.0 ± 1.4) × 10^3^	(56.0 ± 1.4) × 10^3^
MPN	T_0_	Natural sediment^a^	(22.5 ± 0.7) × 10^3^	(22.0 ± 1.4) × 10^3^	(22.5 ± 0.7) × 10^3^	(22.5 ± 0.7) × 10^3^	(22.5 ± 0.7) × 10^3^
	T_80_	TET^c^	(74.0 ± 1.4) × 10^3^	(64.5 ± 0.7) × 10^3^	(65.5 ± 2.1) × 10^3^	(60.0 ± 1.4) × 10^3^	(55.0 ± 5.7) × 10^3^
		PHEN^b^	(60.0 ± 1.4) × 10^3^	(65.5 ± 0.7) × 10^3^	(28.5 ± 0.7) × 10^3^	(24.0 ± 1.4) × 10^3^	(23.5 ± 0.7) × 10^3^
		OIL^b^	(22.5 ± 0.7) × 10^3^	(22.5 ± 0.7) × 10^3^	(22.5 ± 0.7) × 10^3^	(27.5 ± 0.7) × 10^3^	(21.5 ± 0.7) × 10^3^

### 3.4. Microbial abundance in experimental microcosms

At the beginning of the experimental period, microbial abundance values measured in the sediment microcosms corresponded to those recorded in the natural samples. Changes observed after 80 days of incubation under the different experimental conditions, namely tetradecane-enriched microcosms (TET), phenanthrene-enriched microcosms (PHEN), and crude oil-enriched microcosms (OIL), are reported in Table 4 as mean ± standard deviation of duplicate measurements. One-way ANOVA performed on log10-transformed station-level mean values showed significant differences among treatments for total bacterial abundance estimated by DAPI counts, cultivable heterotrophic bacteria estimated by CFU counts, and hydrocarbon-degrading bacteria estimated by the MPN method (p < 0.001 for all parameters). Total bacterial abundance, estimated by DAPI counts, increased significantly in all hydrocarbon-enriched microcosms compared with natural sediments. Mean DAPI values were 4.46 × 10^6^ cells g^−1^ in TET microcosms, 3.13 × 10^6^ cells g^−1^ in PHEN microcosms, and 3.49 × 10^6^ cells g^−1^ in OIL microcosms, whereas natural sediments showed an average value of 2.59 × 10^5^ cells g^−1^. Tukey's post hoc test indicated that TET microcosms showed significantly higher values than both PHEN and OIL microcosms, while no significant difference was observed between PHEN and OIL. Cultivable heterotrophic bacteria showed a different pattern. Mean CFU values were 4.59 × 10^6^ CFU g^−1^ in TET microcosms, 6.31 × 10^5^ CFU g^−1^ in PHEN microcosms, and 6.78 × 10^6^ CFU g^−1^ in OIL microcosms, compared with 5.48 × 10^6^ CFU g^−1^ in natural sediments. Tukey's post hoc test showed that PHEN microcosms had significantly lower CFU values than natural sediments, TET, and OIL microcosms, whereas no significant differences were observed among natural sediments, TET, and OIL microcosms. Hydrocarbon-degrading bacteria, estimated by the MPN method, increased markedly after hydrocarbon enrichment. Mean MPN values were 6.38 × 10^4^ MPN g^−1^ in TET microcosms, 3.27 × 10^3^ MPN g^−1^ in PHEN microcosms, and 2.33 × 10^4^ MPN g^−1^ in OIL microcosms, whereas natural sediments showed an average value of 2.24 × 10^2^ MPN g^−1^. Tukey's post hoc test indicated significant differences among all treatments, with the highest values observed in TET microcosms, followed by OIL, PHEN, and natural sediments. Overall, these results indicate that hydrocarbon enrichment promoted the increase of bacterial populations able to grow under hydrocarbon-selective conditions, with tetradecane showing the strongest enrichment effect on both total bacterial abundance and hydrocarbon-degrading bacteria.

### 3.5. Isolation and taxonomical characterization (16S DNA sequencing)

**Figure 3.**
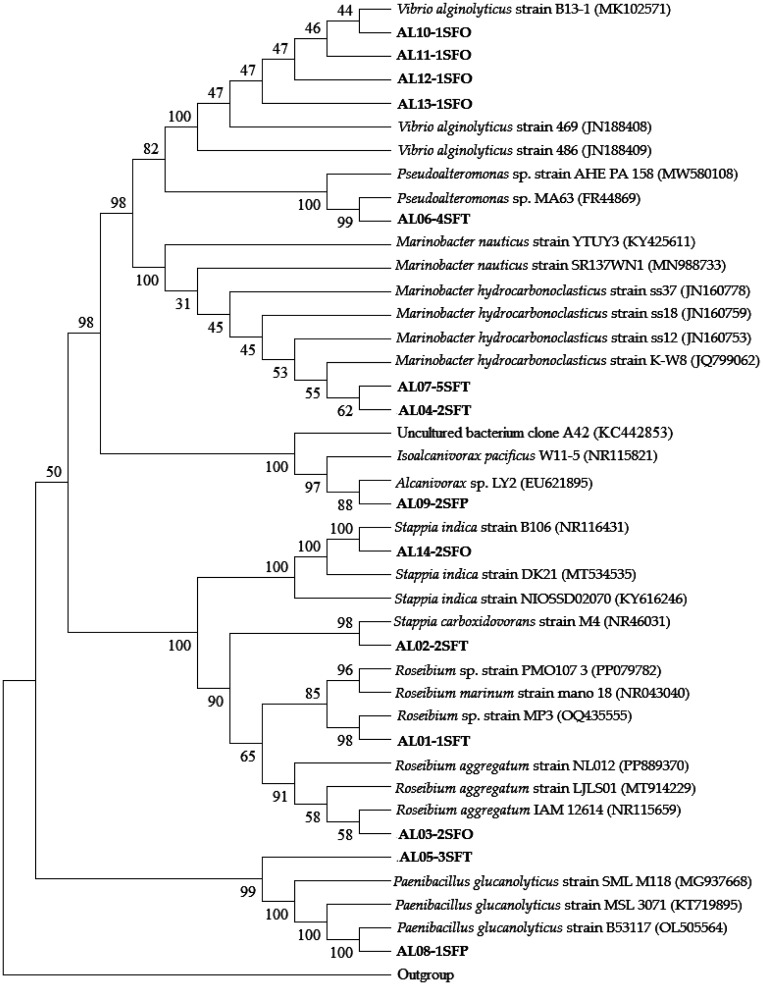
Rooted phylogenetic tree clustered by maximum likelihood union showing the affiliation of partial sequences of the bacterial 16S rRNA gene to the closest sequences of members of different bacterial clusters. Isolates obtained in the present study are reported in bold. The percentages of 1000 bootstrap resampling that supported the branching orders in each analysis are indicated at or near the relevant nodes (only 50% P values are shown). A sequence from an uncultured archaeon clone, *Methanococcus jannaschii* (M59126), was used in the analysis and indicated in the figure as “Outgroup”.

A total of 25 bacterial strains, with different colony morphologies, were isolated from the enrichment cultures. Particularly, 10 bacteria were isolated from the TET microcosms, and 6 and 8 bacteria were isolated from the PHEN and OIL systems, respectively. However, 9 isolates were not included in the taxonomic analysis because sequencing produced low-quality or mixed chromatograms, preventing reliable taxonomic assignment. The molecular characterization of the isolates was carried out by amplifying and partially sequencing the 16S rRNA gene (approximately 1400 bp); the sequences were compared to the database of known 16S rRNA sequences (BLAST). Identical gene sequences were pooled, and only one representative isolate was selected from each group (Table 5). The phylogenetic analysis revealed the distribution of the selected isolates within three taxonomic groups, Gammaproteobacteria, Bacilli, and Alphaproteobacteria, and seven different genera, *Vibrio*, *Paenibacillus*, *Isoalcanivorax*, *Stappia*, *Niallia*, *Pseudoalteromonas*, and *Marinobacter*, were identified. Of the isolated strains belonging to these genera, only 11 were identified to the level of species. Specifically, four sequences were related to the species *Vibrio alginolyticus* (isolates AL10-1SFO, AL11-1SFO, AL12-1SFO, and AL13-1SFO), two sequences to the species *Marinobacter hydrocarbonoclasticus* (isolates AL04-2SFT and AL07-5SFT), one sequence showed similarity to the species *Paenibacillus glucanolyticus* (isolate AL08-1SFP), another with the species *Niallia circulans* (isolate AL05-3SFT), one more with *Roseibium aggregatum* and *R. marinum* (isolates AL03-2SFT and AL01-1SFT, respectively), another with *Isoalcanivorax pacificus* (isolate AL09-2SFP), and finally with *Stappia indica* and *S. carboxidovorans* (isolates AL14-2SFO and AL02-2SFT, respectively) (Figure 3). The bacterial isolates have been deposited in the GenBank database, and the accession numbers reporting their origin and sequences are reported in Table 5.

**Table 5.** Closest relatives of the 16S rRNA gene sequences of the bacterial strains isolated from the different experimental microcosms.

Microcosms		Data isolates
Station	Carbon Sources	Code	Closest hit	Accession N.	ID,%
AL-01	TET	AL01-1SFT	*R. marinum* strain mmo18	PV639156	98.94
AL-02	TET	AL02-2SFT	*S. carboxidovorans* strain M4	PV639157	99.52
AL-02	TET	AL03-2SFT	*R. aggregatum* strain LJLSO1	PV639158	99.41
AL-02	TET	AL04-2SFT	*M. hydrocarbonoclasticus* strain ss30	PV639159	98.28
AL-03	TET	AL05-3SFT	*N. circularis* strain K58-58	PV639160	100.00
AL-04	TET	AL06-4SFT	*Ps*. sp. strain MB_DO_Iso37-Premixed_E01	PV639161	99.36
AL-05	TET	AL07-5SFT	*M. hydrocarbonoclasticus* strain ss18	PV639162	98.34
AL-01	PHEN	AL08-1SFP	*Pa. glucanolyticus* strain B 531/17 16S	PV639163	99.91
AL-02	PHEN	AL09-2SFP	*I. pacificus* strain W11-5	PV639164	99.67
AL-01	OIL	AL10-1SFO	*V. alginolyticus* strain B2-2 16S	PV639165	99.90
AL-01	OIL	AL11-1SFO	*V. alginolyticus* strain V5 16S	PV639166	99.90
AL-01	OIL	AL12-1SFO	*V. alginolyticus* strain B 13-1	PV639167	99.90
AL-01	OIL	AL13-1SFO	*V. alginolyticus* strain B 13-1	PV639168	100.00
AL-02	OIL	AL14-2SFO	*S. indica* strain NIOSSD020#70	PV639169	99.90

### 3.6. Emulsifying activity (%, E_24_)

**Figure 4.**
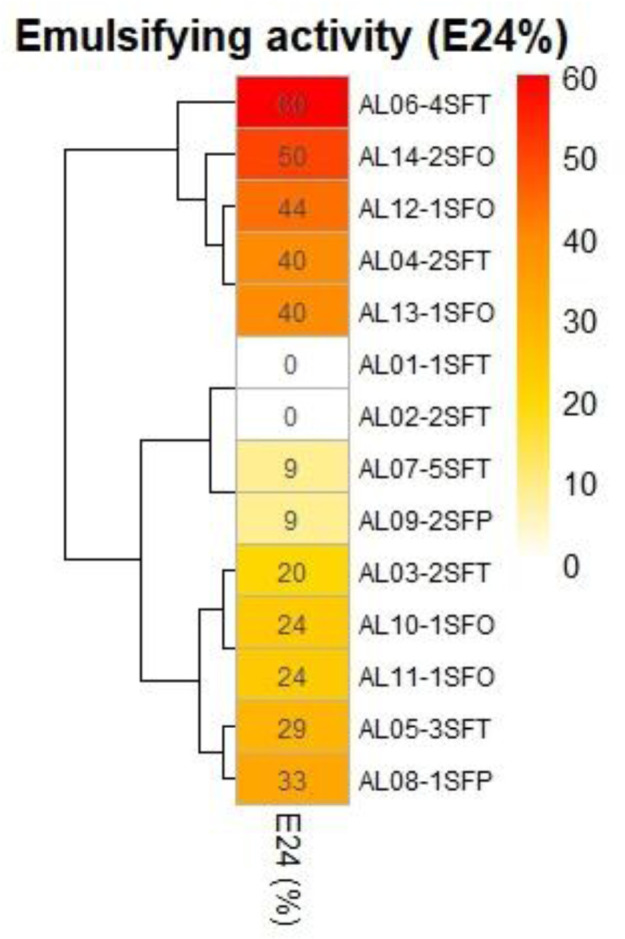
Clustered heat map of the emulsifying activity (E_24_, %) measured in the bacterial isolates obtained from hydrocarbon-enriched microcosms. The color gradient represents E_24_ values expressed as percentages, with higher color intensity indicating higher emulsifying activity. Hierarchical clustering was performed based on E_24_ values to group isolates according to their emulsification capacity.

Among the bacterial isolates, those that showed the highest percentage values of emulsifying activity were the strains AL06-4SFT (60%), AL14-2SFO (50%), and AL12-1SFO (44%). The clustered heat map grouped the isolates according to their emulsifying activity, separating strains with high E_24_ values, such as AL06-4SFT, AL14-2SFO, and AL12-1SFO, from isolates showing low or no emulsifying activity. In general, high percentages were also recorded for strains AL04-2SFT and AL13-1SFO, reaching percentages of emulsification of 40% (Figure 4).

## 4. Discussion

The survey area, Lake Faro, represents a meromictic lake with peculiar characteristics because it is strongly influenced by the adjacent Strait of Messina, which communicates with it through channels, and it is subject to high anthropogenic pressure and environmental variability [33] due, in part, to the shellfish farming activity that this environment hosts. High seasonal variability rather than spatial variability characterizes the entire Cape Peloro area, with low prokaryotic abundance and microbial metabolism in Lake Faro compared to Ganzirri, and salinity values similar to those found in marine environments [53]. The environmental context of Lake Faro is relevant for interpreting the occurrence of hydrocarbon-degrading bacteria in the investigated sediments. The sampling stations were located in shallow sediments of a brackish meromictic basin influenced by hydrological exchanges with adjacent marine systems and by local anthropogenic activities [33]–[36]. In the present study, chemical analyses showed no detectable HCs > 12 in surface sediments, whereas PAHs were detected only at low concentrations. Nevertheless, the enrichment experiments showed a marked increase in hydrocarbon-degrading bacteria after the addition of selected hydrocarbons, particularly tetradecane and crude oil. This response suggests that the sediment microbial fraction included hydrocarbon-responsive populations that were not dominant or functionally evident under natural conditions but became detectable under selective enrichment. This pattern is consistent with the concept of microbial seed banks, where rare or dormant microbial populations can persist in the environment and respond when suitable substrates or environmental conditions become available [32]. This view is also compatible with the variable culturability of environmental microorganisms, since low-activity or dormant cells may be recovered only under specific enrichment conditions. Resuscitation-promoting mechanisms have been shown to accelerate the enrichment of difficult-to-culture dechlorinating microbial populations, while exposure to high concentrations of aromatic contaminants can induce viable but non-culturable states in pollutant-degrading microorganisms [54],[55]. Therefore, in the present study, the term “silent” should be interpreted as referring to a cultivable hydrocarbon-responsive fraction revealed by enrichment, rather than as direct evidence of a specific physiological state. In this perspective, the hydrocarbon-degrading bacteria detected in Lake Faro sediments should not be interpreted as evidence of active hydrocarbon contamination but rather as an intrinsic functional reservoir potentially involved in natural attenuation and ecological resilience. The enrichment patterns observed in the microcosms indicate that the cultivable sediment-associated bacterial fraction responded differently to the tested hydrocarbon substrates. Tetradecane-amended microcosms showed the strongest increase in total bacterial abundance and MPN values, suggesting a marked selection of bacteria able to grow under aliphatic hydrocarbon-enriched conditions. Crude oil microcosms also supported a clear increase in hydrocarbon-degrading bacteria, consistent with the complex composition of crude oil as a mixture of aliphatic and aromatic compounds. In contrast, phenanthrene-amended microcosms showed a lower enrichment response, particularly for the cultivable heterotrophic fraction, suggesting that this aromatic substrate may have exerted stronger selective pressure on the sediment bacterial community.

After 80 days of experimentation and quantitative analysis, as well as taxonomic identification, a total of 16 bacterial strains belonging to the groups of Alphaproteobacteria, Gammaproteobacteria, and Bacilli were identified. As previously reported [56], Proteobacteria (to which Alphaproteobacteria and Gammaproteobacteria belong) are the most abundant phylum of bacteria commonly observed in the waters and sediments of lakes or even soil and marine environments. In our experimental study, the bacterial strains *R. aggregatum* (isolate AL03-2SFT), *R. marinum* (isolate AL01-1SFT), *S. carboxidovorans* (isolate AL02-2SFT), and *S. indica* (isolate AL14-2SFO) isolated from tetradecane-enriched and petroleum-enriched microcosms, belonged to Alphaproteobacteria. Specifically, the species *Roseibium* is ubiquitous in different environments; in fact, it has been isolated from saline lakes [57], marine sediments [58], and freshwater [59]. The genus *Stappia* is generally found associated with various marine environments [48] and ocean sediments [60] but also with dinoflagellates [61] and various marine invertebrates [62],[63]. Enrichments with strains of *S. indica*, isolated from deep seawater, were reported to degrade petroleum hydrocarbons in pure culture; moreover, this species was detected in petroleum-contaminated marine environments [64]. Conversely, *S. carboxidovorans*, isolated as strain M4 from the alga *Ascophyllum nodosum*, displays a broad metabolic spectrum, growing on various organic carbon sources (such as sugars, organic acids, and amino acids) but not on alcohols or aromatic compounds [65]. In our enriched microcosms, the bacterial strains *M. hydrocarbonoclasticus* (strains AL04-2SFT and AL07-5SFT), *Pseudoalteromonas* sp. (strains AL06-4SFT), *I. pacificus* (strain AL09-2SFP), and *V. alginolyticus* (strains AL10-1SFO, AL11-1SFO, AL12-1SFO, AL13-1SFO, and AL14-1SFO) isolated from the microcosms TET (*M. hydrocarbonoclasticus*, *Pseudoalteromonas* sp.), PHEN (*I. pacificus*), and OIL (*V. alginolyticus*), all belonged to the Gammaproteobacteria. Members of the genus *Marinobacter* are well-known oil-degrading bacteria; this taxon is metabolically versatile, as it can utilize a wide range of carbon substrates. including aliphatics and PAHs [66],[67]. Moderate amounts of oil degradation by *M. hydrocarbonoclasticus* (responsible for the removal of 36% of the chloroform-extractable oil fraction) and the involvement of this species in oil degradation in contaminated sediment were reported in the literature [68]. Like *Marinobacter*, *Vibrio* spp. also have a versatile metabolism that allows them to use different carbon substrates. As reported by Isiodu [69], *V. alginolyticus* G19 can degrade as much as 90% diesel when included in bacterial consortia, and it has also been isolated from brackish water contaminated with petroleum. *Pseudoalteromonas* sp. is a strictly marine genus widespread in the marine environment, belonging to the class Gammaproteobacteria. *Pseudoalteromonas* strains are all Gram-negative, heterotrophic, aerobic bacteria ubiquitous in marine environments, accounting for ~2.6% of surface bacterioplankton along a latitudinal gradient [70] and less than ~1% at a location in the southern North Sea [71]. Like the previously mentioned species, *Pseudoalteromonas* spp. also have a versatile metabolism and can utilize different carbon sources, as reported in a study by Chronopoulou [72], in which *Pseudoalteromonas* NS50 strains were grown using linear alkanes (tetradecane), branched alkanes, and PAHs. *Isoalcanivorax pacificus* is a member of one of the three *Alcanivorax* clades. Unlike the *Alcanivorax* group, whose physiological and metabolic characteristics are widely studied as the basis for the application of environmental recovery techniques, those of *Isoalcanivorax* remain as yet little explored [73]. Finally, of the isolates identified taxonomically from our microcosms, two isolates (strains AL08-1SFP and AL08-1SFP) belonging to the *Bacillus* class were identified as *P. glucanolyticus* and *N. circulans*, respectively. *P. glucanolyticus* was isolated by Ghafari [74] from a polluted marine environment, as well as in soils polluted by petroleum hydrocarbons, while *N. circulans* (previously classified as *Bacillus circulans*) showed the ability to degrade lower n-alkanes (C13: 100% degradation; C16: 98.6% degradation; C18: 66.81% degradation; C17: 64.4% degradation) [75]. Overall, the enrichment-based recovery of these isolates, together with their taxonomic affiliation and emulsifying activity, indicates the selection of a cultivable bacterial fraction with hydrocarbon-associated functional traits. Therefore, these isolates are interpreted here as hydrocarbon-responsive bacteria with potential relevance for biodegradation processes. The occurrence of hydrocarbon-responsive bacteria in Lake Faro sediments is consistent with previous studies showing that sedimentary, coastal, and transitional aquatic environments can host bacterial populations with hydrocarbon-degrading potential. In a freshwater lake system, hydrocarbon-degrading consortia including genera such as *Pseudomonas*, *Acinetobacter*, *Stenotrophomonas*, *Aeromonas*, *Sphingobacterium*, *Comamonas*, *Flavobacterium*, and *Enterobacter* were reported, with sediment-associated consortia showing relevant petroleum-hydrocarbon degradation capacity, probably supported by biosurfactant production [76]. In coastal lagoon and river sediments from the Southern Gulf of Mexico, hydrocarbonoclastic bacteria were evaluated for hexadecane biodegradation, showing that lagoon and river-mouth sediments can harbor microbial communities able to degrade aliphatic hydrocarbons [77]. Similarly, Wanapaisan et al. [78] described the synergistic degradation of pyrene by a mangrove sediment-derived bacterial consortium, confirming the role of sediment-associated microorganisms in PAH degradation in coastal transitional environments. More generally, hydrocarbon biodegradation is commonly associated with metabolically versatile bacterial consortia rather than with single bacterial strains, because different members of a consortium may contribute complementary metabolic steps during hydrocarbon transformation [47]. The isolates obtained in the present study, including *Marinobacter hydrocarbonoclasticus*, *Pseudoalteromonas* sp., *Vibrio alginolyticus*, *Stappia indica*, and *Isoalcanivorax pacificus*, therefore agree with the broader evidence that hydrocarbon-enriched conditions can select for bacteria able to respond to aliphatic and/or aromatic substrates. Compared with previous studies mainly focused on contaminated or experimentally amended systems, the present work provides evidence of a cultivable hydrocarbon-responsive fraction in Lake Faro surface sediments, where HCs > 12 were below the quantification limit, and PAH concentrations were low. This supports the hypothesis that these sediments may host a latent functional reservoir able to respond to hydrocarbon enrichment, rather than a bacterial fraction shaped by active hydrocarbon contamination. This study represents the first report of *Isoalcanivorax pacificus* among hydrocarbon-responsive bacterial isolates recovered from Lake Faro sediments.

## 5. Conclusions

The results obtained in this study showed that Lake Faro surface sediments, despite the absence of detectable HCs > 12 and the occurrence of low PAHs concentrations, hosted a cultivable hydrocarbon-responsive bacterial fraction that became detectable after selective enrichment. These findings indicate that Lake Faro sediments host a cultivable hydrocarbon-responsive bacterial fraction that may represent a promising reservoir for future studies aimed at evaluating natural attenuation processes and selecting candidate strains for bioremediation-oriented applications.

## Use of AI tools declaration

The authors declare they have not used Artificial Intelligence (AI) tools in the creation of this article.
